# FEV1 and total Cardiovascular mortality and morbidity over an 18 years follow-up Population-Based Prospective EPIC-NORFOLK Study

**DOI:** 10.1186/s12889-019-6818-x

**Published:** 2019-05-03

**Authors:** Siew-Mooi Ching, Yook-Chin Chia, Marleen A. H. Lentjes, Robert Luben, Nicholas Wareham, Kay-Tee Khaw

**Affiliations:** 1 Department of Family Medicine, Faculty of Medicine and Health Sciences, Universiti Putra Malaysia, Serdang, Malaysia; 2 Malaysian Research Institute on Ageing, Faculty of Medicine and Health Sciences, Universiti Putra Malaysia, Serdang, Malaysia; 3 Department of Medical Sciences, School of Healthcare and Medical Sciences, Sunway University, Bandar Sunway, Malaysia; 4 Department of Public Health and Primary Care, Institute of Public Health, School of Clinical Medicine (K-TK and RL) and the Medical Research Council Epidemiology Unit (NW), University of Cambridge, Cambridge, United Kingdom

**Keywords:** FEV1, Cardiovascular, mortality, morbidity, Population-Based, Prospective, Respiratory disease, EPIC-NORFOLK

## Abstract

**Background:**

Our study aimed to determine the association between forced expiratory volume in one second (FEV1) and subsequent fatal and non-fatal events in a general population.

**Methods:**

The Norfolk (UK) based European Prospective Investigation into Cancer (EPIC-Norfolk) recruited 25,639 participants between 1993 and 1997. FEV1 measured by portable spirometry, was categorized into sex-specific quintiles. Mortality and morbidity from all causes, cardiovascular disease (CVD) and respiratory disease were collected from 1997 up to 2015. Cox proportional hazard regression analysis was used with adjustment for socio-economic factors, physical activity and co-morbidities.

**Results:**

Mean age of the population was 58.7 ± 9.3 years, mean FEV1 for men was 294± 74 cL/s and 214± 52 cL/s for women. The adjusted hazard ratios for all-cause mortality for participants in the highest fifth of the FEV1 category was 0.63 (0.52, 0.76) for men and 0.62 (0.51, 0.76) for women compared to the lowest quintile. Adjusted HRs for every 70 cL/s increase in FEV1 among men and women were 0.77 (*p* < 0.001) and 0.68 (*p* < 0.001) for total mortality, 0.85 (*p*<0.001) and 0.77 (*p*<0.001) for CVD and 0.52 (*p* <0.001) and 0.42 (*p* <0.001) for respiratory disease.

**Conclusions:**

Participants with higher FEV1 levels had a lower risk of CVD and all-cause mortality. Measuring the FEV1 with a portable handheld spirometry measurement may be used as a surrogate marker for cardiovascular risk. Every effort should be made to identify those with poorer lung function even in the absence of cardiovascular disease as they are at greater risk of total and CV mortality.

## Background

It is known that patients with chronic obstructive pulmonary disease and those with low forced expiratory volume in 1 second (FEV1) have increased incidence of cardiovascular disease (CVD) [1, 2] and all-cause mortality [3, 4]. However, less is known about lung function and cardiovascular mortality and morbidity in a normal population, particularly among the non-smokers and ex-smokers. Although many population based studies of lung function have adjusted for smoking habits, body mass index and co-morbidities, few studies have considered the role of socio-economic factors and physical inactivity in relation to lung function and total mortality [5]. This study aimed to examine whether FEV1 at baseline is a risk factor for mortality and morbidity of cardiovascular and respiratory diseases in a population-based cohort over an 18-year period.

## Methods

### Participants

The study population was based in the Norfolk region, United Kingdom (UK). A total of 77,630 men and women aged between 40–79 were selected from age-sex registers of general practices and were invited by post to participate in the baseline survey [6]. The participants were part of the European Prospective Investigation into Cancer (EPIC) study [6].

Eligibility criteria

1. Adults aged 40-79 years from age-sex registers of general practices

Exclusion criteria

1. Those who have ischaemic heart disease, stroke or cancer at baseline.
2. Non-responders to the postal invitation to join the study.

This large multicentre prospective general population study was originally designed not only to investigate the relationship between diet, cancer, and chronic diseases [7], but it also explores exposures other than diet, and outcomes other than cancer as part of the larger study [6]. Out of 30,445 subjects who consented to participate in the study, 25,639 men and women actually attended a baseline health check between 1993-1997 [6]. Ethics approval was obtained from the Norfolk Ethics Committee (Rec Ref: 98CN01) and all participants gave informed written consent prior to the study.

### Data collection

#### FEV1 measurement

All subjects were instructed by trained nurses to perform spirometry measurements using a portable electronic turbine spirometer (Micro Medical, Rochester, UK) following a standard protocol. Two measurements were recorded for each participant and the higher of the two values for FEV1 was entered for analysis. The reproducibility of FEV1 was 2.2%. A previous study reported that this device was accurate and comparable to the Vitalographspirometer [8] and the reproducility of FEV1 differed by only 2.2%. Calibration was performed regularly on a weekly basis to ensure the accuracy and precision of both equipment and personnel [6]. Only FEV1 is reported here. Although the handheld spirometry device measures both FEV1 and forced vital capacity (FVC), the FVC measures did not add any extra information [9].

#### Anthropometric, lifestyle and socio-economic measurements

Trained nurses measured participants’ height and weight with patients wearing light clothing and without shoes. They used a stadiometer to measure height to the nearest 0.1 cm, and Salter scale for weight to the nearest 100 g. We computed the Body Mass Index (BMI) as weight in kilograms per square meter height (kg/m2). Blood Pressure (BP) was recorded using an Accutorr non-invasive oscillometric blood pressure monitor (Datascope Medical, Huntingdon, United Kingdom) after the participant had been seated for 5 minutes. The mean of 2 readings was entered for analysis. Non-fasting blood samples were taken. Plasma concentration of total cholesterol was measured with the RA 1000 Technicon analyser (Bayer Diagnostics, Basingstoke) and the plasma vitamin C concentration was estimated by using a fluorometric assay within a week of sampling [10, 11].

For data collection, all participants provided their information via several self-administered questionnaires. The questionnaires included sociodemographic data, a comorbidities section as well as a health and lifestyle questionnaire (HLQ). The diagnosis of chronic diseases like diabetes, hypertension, ischaemic heart disease, stroke, cancer, asthma, and bronchitis were recorded as present when participants answered “yes” to question “Has a doctor ever told you that you have any of the following conditions?” Smoking status based on the health and lifestyle questionnaire was classified as current, former, or never smokers. Current smokers were defined as those currently smoking cigarettes, former smoker as being a smoker previously and non-smokers were those who never smoked. Alcohol status was based on the estimated total alcohol consumption in a week as reported in the health and lifestyle questionnaire and categorized as current drinker, former drinker, or non-drinker [6].

Social class was categorised into 5 groups based on the Registrar General’s occupation-based classification scheme. Social class I: professional jobs, social class II: secretarial and technicians. Social class III: non-manual and manual skilful workers. Social class IV: partially skilful workers; social class V: unskilful manual workers. We re-categorised social class into non-manual (social class I–III non-manual) and manual (social class III manual, IV, and V) for our analysis [12].

We classified physical activity into four levels (inactive, moderately inactive, moderately active and active) using a self-reported physical activity index, validated with heart-rate monitoring [13]. We reclassified the participants as physically inactive (level I) or not physically inactive (level II-IV) [14].

Educational status was classified into four groups: degree, A-level, O-level, and below O level. O-level is applied for those participants who had completed their schooling at the age of 15 years whereas A-level refers to participants who had completed their schooling at the age of 17 years. We also reclassified educational level as “at least O-level” (which consist of those with educational achievement of either O-level, A-level or degree) and “no qualifications” [12]. Non-fasting blood samples were obtained by venepuncture as previously described [6].

#### Outcome

The outcomes of this study are defined as having cardiovascular or respiratory events. Cardiovascular events are defined as fatal or non-fatal myocardial infarction, angina, heart failure and stroke. Respiratory disease events are defined as fatal and non-fatal asthma, bronchitis and chronic obstructive pulmonary disease.

#### Outcome ascertainment

All fatal events occurring between 1993 and 31^st^ January 2015 (an average of 18 years of follow-up) were captured by the Office for National Statistics, United Kingdom. Both fatal cardiovascular (myocardial infarction and stroke) and fatal respiratory disease (bronchitis, emphysema and asthma) were identified for analysis. Non-fatal cardiovascular and respiratory events up to March 2009 were identified from the data linkage with East Norfolk Health Authority database using participant’s unique National Health Service number.

#### Follow-up

This was part of the 25-year follow up of the EPIC-Norfolk study. The study was divided into 5 phases. The first phase was in 1993-1997, second 1998-2000, third 2004-2011, fourth 2012-2016 and fifth 2016-2018. The follow-up was done during each phase. There were 25,639 participants in the initial phase and by fifth phase, the participants were 5696. Blood, urine test and questionnaire were collected during the follow-up. In this study, the follow-up was done from 1993 to 2015 in the second, third and fourth phase in this study. Baseline FEV1 was done and the vital status of the participants was ascertained at the fourth follow up.

#### Statistical analysis

All statistical analyses were performed using the Statistical Package for Social Sciences (SPSS version 22). A total of 25,639 participants were entered this analysis. We excluded 596 patients who did not have an FEV1 measurement. Out of the remaining 25,043 subjects, we further excluded subjects who had a baseline self-report of myocardial infarction (*n*=807) or stroke (n=363) or cancer (n=1410) leaving a total of 22,662 eligible participants (10,168 men and 12,494 women)

Participants were divided into sex-specific FEV1 quintiles, and subsequent analyses were sex-specific. We studied the risk factors distributions among men and women by FEV1 quintiles. Continuous data are described using mean and standard deviation when the distribution was normal; when the data had a skewed distribution, median and interquartile range (25-75th percentile) were used. Categorical data are reported as percentage and differences tested using Chi-square test or Fisher exact tests.

We used Cox proportional hazards model to determine the Hazard Ratio (HRs) by quintiles of all-cause and disease-specific mortality as well as total fatal and non-fatal incident cardiovascular and respiratory diseases based on FEV1 quintiles after age adjustment in model 1. This was followed by model 2 with additional covariables of education level, social class, self-reported diabetes, respiratory disease, smoking status, alcohol consumption, physical activity, height, BMI, systolic blood pressure, plasma vitamin C concentration and plasma total cholesterol level. We used regression modelling to test for trend.

We also examined HRs in a subgroup analysis stratified by sex and adjusted for age group, BMI, social class, smoking status, physical activity, plasma vitamin C level and after the exclusion of those with any history of respiratory diseases (prevalent bronchitis/ emphysema and asthma) and those who died < 5 years of follow-up. Statistical significance is defined as p-value < 0.05. We also calculated regression coefficients with 95 percent confidence intervals.

## Results

A total of 22,662 participants were entered analysis and are described in Table 1. There were 44.9% men (n=10,168). Mean age of men and women was 58.9 years old and 58.5 years old respectively. Mean FEV1 was 294±74 cL/s for men and 214 ± 52 cL/s for women. Total all-cause mortality events were 5854 over the 18 years. CVD mortality events were 957 (9.4%) for men and 798 for women (6.4%) and respiratory disease mortality events were 267 in men (2.6%) and 311 in women (2.5%). There were 3307(32.5%) and 3378(14.5%) total cardiovascular events and 1391(13.7%) and 1844(14.8%) respiratory disease events for men and women respectively. Mean baseline FEV1 for men who died was 248cL/s and 310cL/s for men alive at end of follow-up (p<0.001); 180cL/s for women who died and 223cL/s for women who were alive (p<0.001) respectively. Mean baseline age for men who died was 66.3 years and 55.8 years for men alive at end of follow-up; 66.6 years for women who died and 56.2years for women alive respectively. The baseline FEV1 was lower among the older participants compared to the younger participants for both genders, and the baseline FEV1 was also significantly lower among those who died prior to the end of the study compared to those still alive for both male (*p*<0.001) and female (*p*<0.001).

**Table 1:** Characteristics of 10,168 men and 12,494 women aged 40-79 years without prevalent heart disease, stroke, or cancer, by quintile of FEV1 in the EPIC-Norfolk, United Kingdom, recruited between 1993-1997

Clinical variables	Unit	Respiratory function FEV1 category (cL/s)					P*
Quintile		<229	229 to <272	272 to < 309	309 to < 353	≥ 353	
Men (%, n)	100, 10168	18.3, 1857	19.3, 1959	20.5, 2087	20.6, 2097	21.3, 2168	
Mean respiratory function FEV1 (cL/s)	293.7±73.9	184.1± 39.6	252.0±12.1	291.2±10.9	330.7±12.7	392.0±31.5	<0.001
Mean age (year)	58.9±9.3	66.0± 7.9	62.9±8.5	59.3±8.5	56.0±7.8	51.9±6.2	<0.001
Mean Height (cm)	174.2±6.7	170.7±6.5	1712.0±6.2	173.7±6.1	175.5±6.0	178.3±5.8	<0.001
Mean systolic BP (mm Hg)	137.2±17.5	142.8± 19.0	140.8±17.7	137.5±17.8	134.9±15.8	131.1±14.9	<0.001
Mean alcohol (units/week)	10.4±11.9	9.4± 11.9	10.1±12.2	9.9±11.5	11.0±12.1	11.5±11.6	0.411
Mean BMI (kg/m2)	26.5±3.3	26.8± 3.7	27.0±3.3	26.5±3.3	26.3±3.1	26.0±3.1	<0.001
Mean plasma vitamin C (umol/l)	47.4±18.7	42.7± 20.0	45.3±18.2	47.9±18.6	49.1±17.6	51.0±18.2	<0.001
Mean serum cholesterol (mmol/L)	6.0±1.1	6.1±1.1	6.1±1.1	6.1±1.0	6.1±1.1	5.9±1.1	0.897
Physically inactive [% (n)]	29.3(2982)	43.7 (812)	33.2(650)	27.4(571)	24.1(506)	20.4(443)	<0.001
Manual social class [% (n)]	41.6(4160)	48.3 (870)	45.6(877)	42.8(877)	38.9(805)	34.1(731)	<0.001
No educational qualification [% (n)]	29.5(3001)	44.2 (819)	34.6(678)	29.7(620)	24.9(521)	16.8(363)	<0.001
Current smokers [% (n)]	12.5(1261)	17.7 (327)	12.8(249)	13.2(273)	11.6(242)	7.9(170)	<0.001
History of diabetes [% (n)]	2.7 (271)	4.0 (74)	4.0(79)	2.6(54)	2.1(45)	0.9(19)	<0.001
History of asthma [% (n)]	7.7(787)	17.1 (318)	7.9(154)	6.2(129)	4.9 (102)	3.9(84)	<0.001
History of bronchitis [% (n)]	8.7(886)	16.2 (300)	8.1(159)	6.9(143)	6.7(140)	6.6(144)	<0.001
Clinical variables	Unit	Respiratory function FEV1 category (cL/s)					P*
Quintile		<170	170 to <200	200 to < 227	227 to < 257	≥ 257	
Women (%, n)	100, 12494	19.6, 2449	19.3, 2413	20.2, 2528	20.4, 2555	20.4, 2549	
Mean respiratory function FEV1(cL/s)	214.4±52.0	139.9 ± 26.6	186.3±8.7	213.9±7.8	241.7±8.5	285.4±23.0	<0.001
Mean age (year)	58.5±9.3	66.1± 7.7	62.6±8.5	58.4±8.3	54.8±7.4	51.2±6.1	<0.001
Mean Height (cm)	161.0±6.2	157.6±6.0	159.2±5.5	160.8±5.5	162.2±5.5	165.1±5.5	<0.001
Mean systolic BP(mm Hg)	133.6±18.8	142.5± 19.9	137.9±19.3	133.3±17.4	129.5±16.9	125.4±15.6	<0.001
Mean alcohol (units/week)	4.5±5.7	3.7±5.3	3.9±5.1	4.5±5.8	4.9±5.9	5.5±6.1	<0.001
Mean BMI (kg/m2)	26.2±4.3	26.9±4.5	26.8±4.6	26.3±4.3	25.9±4.2	25.1±3.8	<0.001
Mean vitamin C (umol/l)	58.6±19.9	54.5±21.0	57.6±20.3	58.8±19.9	59.9±19.6	61.8±17.8	<0.001
Mean cholesterol (mmol/L)	6.3±1.2	6.7±1.2	6.5±1.2	6.4±1.2	6.2±1.2	5.8±1.1	<0.001
Physical inactive [% (n)]	29.5(3687)	46.3 (1134)	36.1 (871)	26.3 (664)	22.2 (568)	17.7 (450)	0.005
Manual social class [% (n)]	38.9(4738)	44.8 (1045)	42.6 (997)	39.7 (977)	35.6 (893)	32.7 (826)	<0.001
No educational qualification [% (n)]	41.7(5205)	58.0 (1419)	49.9 (1204)	42.9 (1083)	34.6 (885)	24.1 (614)	<0.001
Current smokers [% (n)]	11.5(1426)	14.1 (341)	12.0 (286)	11.0 (275)	11.3 (287)	9.3 (237)	<0.001
History of diabetes [% (n)]	1.4(180)	2.2 (55)	2.1(50)	1.5 (38)	0.9 (23)	0.5 (14)	<0.001
History of asthma [% (n)]	9.0(1120)	16.5 (404)	8.7(209)	7.7 (194)	6.4 (164)	5.8 (149)	<0.001
History of bronchitis [% (n)]	9.5(1190)	13.9. (340)	9.4 (226)	8.6 (217)	8.3 (212)	7.7 (195)	<0.001

*P-value is the association between FEV1 in category with the clinical variables based on ANOVA or chi-square test

†BP blood pressure; BMI, body mass index; SD, standard deviation; FEV1, forced expiratory volume in 1 second; § Weight (kg)/height (m)2

# Self-reported doctor-diagnosed condition

Comparing the top quintile of FEV1 with the bottom quintile, FEV1 was approximately one and a half times higher for both men and women. Men and women in progressively higher FEV1 quintiles were younger, had lower systolic blood pressure and anthropometric measures, a lower prevalence of diabetes, asthma and bronchitis, fewer current smokers and fewer persons of social class III, IV and V, a higher education qualification, higher plasma vitamin C and were more physically active. Overall, cardiovascular disease risk factors were significantly lower in those with better lung function (FEV1) (p<0.001).

Men who were excluded because of missing respiratory function values or having either myocardial infarction, stroke or cancer at baseline (n = 1203) were older (65 years vs. 59 years *p* < 0.001), slightly heavier (BMI 26.9 kg/m^2^ vs. 26.5 kg/m^2^ p<0.001) and slightly shorter (173 cm vs. 174 cm *p*<0.001) compared to men who were included in the current analysis. Women who had been excluded (*n* = 1253) were older (63 years vs. 59 years *p* < 0.001) and slightly shorter (160cm vs. 161cm *p*<0.001), but there was no difference in BMI (26.4 kg/m^2^ vs. 26.2 kg/m^2^ *p* = 0.07), when they were compared to women included in the analysis.

Mortality percentage from 1993 to 2015 and HRs by FEV1 quintile by cause are shown in Table 2. HRs for incident total cardiovascular diseases, myocardial infarction, stroke, and respiratory diseases adjusted first for age and stratified by sex, and further multivariable adjusted as described previously are also shown in Table 2.

**Table 2:** Hazard ratios by FEV category for mortality by cause and incidence for cardiovascular disease and respiratory disease in 22662 men and women in the EPIC-Norfolk 1993-2015

	Respiratory function FEV1 category (cL/s)						
Quintile	<229	229 to <272	272 to < 309	309 to < 353	≥ 353	*HR(95% CI) per 70 cL/s increase*	P-linear trend
Men							
Deaths: all causes % (n)	35.8 (1108)	25.7 (794)	18.7 (579)	12.7 (394)	7.1 (221)		<0.001
HR (95% CI) ^a^	1	0.69(0.63,0.75)	0.60(0.54,0.66)	0.55(0.49,0.62)	0.48(0.41,0.56)	0.72(0.69,0.75)	<0.001
HR (95% CI) ^b^	1	0.74(0.67,0.82)	0.69(0.61.77)	0.66(0.58,0.76)	0.63(0.52,0.76)	0.77(0.73,0.81)	<0.001
Deaths from cardiovascular causes % (n)	37.5 (359)	29.2 (279)	16.3 (156)	12.1 (116)	4.9 (47)		<0.001
HR (95% CI) ^a^	1	0.77(0.66,0.9)	0.54(0.44,0.65)	0.57(0.46,0.72)	0.39(0.28,0.54)	0.70(0.65,0.76)	<0.001
HR (95% CI) ^b^	1	0.89(0.75,1.06)	0.67(0.54,0.84)	0.75(0.58,0.97)	0.64(0.44,0.92)	0.80(0.73,0.88)	<0.001
Deaths from myocardial infarction causes % (n)	35.1 (191)	29.5 (161)	15.2 (83)	14.1 (77)	6.1 (33)		<0.001
HR (95% CI) ^a^	1	0.84(0.68,1.04)	0.54(0.41,0.70)	0.71(0.53,0.94)	0.50(0.33,0.75)	0.75(0.68,0.83)	<0.001
HR (95% CI) ^b^	1	0.99(0.78,1.26)	0.70(0.52,0.94)	0.97(0.70,1.35)	0.90(0.57,1.42)	0.88(0.78,1.00)	0.05
Deaths from stroke causes % (n)	33.7 ( 68)	31.7 (64)	19.8 (40)	12.3 (25)	2.5 (5)		<0.001
HR (95% CI) ^a^	1	0.91(0.65,1.28)	0.72(0.48,1.08)	0.66(0.41,1.08)	0.24(0.09,0.61)	0.73(0.62,0.86)	0.027
HR (95% CI) ^b^	1	0.97(0.66,1.42)	0.78(0.49,1.23)	0.78(0.45,1.37)	0.38(0.14,1.04)	0.78(0.64,0.95)	0.015
Deaths from respiratory disease causes %(n)	62.4 (184)	17.6 (52)	12.5 (37)	5.1 (15)	2.4 (7)		<0.001
HR (95% CI) ^a^	1	0.27(0.20,0.37)	0.24(0.17,0.35)	0.14(0.08,0.25)	0.12(0.05,0.26)	0.32(0.28,0.36)	<0.001
HR (95% CI) ^b^	1	0.33(0.23,0.46)	0.29(0.19,0.43)	0.20(0.11,0.35)	0.19(0.08,0.43)	0.35(0.30,0.41)	<0.001
Cardiovascular disease events %(n)	28.5 (1028)	23.9 (863)	21.1 (760)	15.9 (576)	10.6 (384)		<0.001
HR (95% CI) ^a^	1	0.78(0.71,0.85)	0.73(0.66,0.80)	0.64(0.57,0.71)	0.51(0.45,0.58)	0.80(0.77,0.83)	<0.001
HR (95% CI) ^b^	1	0.82(0.74,0.91)	0.81(0.72,0.90)	0.73(0.64,0.82)	0.63(0.54,0.73)	0.85(0.81,0.89)	<0.001
Myocardial infarction events % (n)	30.6(528)	25.0(431)	19.7 (340)	15.9 (275)	8.8 (152)		<0.001
HR (95% CI) ^a^	1	0.78(0.69,0.89)	0.66(0.58,0.76)	0.63(0.54,0.74)	0.42(0.34,0.51)	0.77(0.73,0.81)	<0.001
HR (95% CI) ^b^	1	0.85(0.74,0.98)	0.78(0.67,0.91)	0.79(0.66,0.95)	0.58(0.46,0.73)	0.85(0.79,0.91)	0.001
Stroke events % (n)	34.7 (157)	28.0 (127)	16.8 (76)	12.8 (58)	7.7 (35)		<0.001
HR (95% CI) ^a^	1	0.86(0.68,1.08)	0.62(0.47,0.82)	0.66(0.48,0.92)	0.64(0.43,0.97)	0.79(0.71,0.88)	0.008
HR (95% CI) ^b^	1	0.98(0.75.1.28)	0.78(0.57,1.08)	0.86(0.59,1.26)	0.99(0.96,1.02)	0.90(0.79,1.03)	0.13
Respiratory disease events %(n)	41.3 (669)	22.0 (357)	16.7 (271)	11.5 (186)	8.5 (138)		<0.001
HR (95% CI) ^a^	1	0.46(0.40,0.52)	0.34(0.29,0.39)	0.25(0.21,0.30)	0.21(0.17,0.26)	0.51(0.48,0.54)	<0.001
HR (95% CI) ^b^	1	0.48(0.41,0.55)	0.37(0.32,0.44)	0.28(0.23,0.33)	0.26(0.18,0.28)	0.52(0.49,0.55)	<0.001
	Respiratory function FEV1 category (cL/s)						
Quintile	<170	170 to <200	200 to < 227	227 to < 257	≥ 257	*HR(95%CI) per 70 cL/s increase*	P-linear trend
Women							
Deaths: all causes % (n),2758	42.1 (1161)	25.0 (688)	16.6 (459)	9.6 (266)	6.7 (184)		<0.001
HR (95% CI) ^a^	1	0.68(0.63,0.75)	0.62(0.55,0.69)	0.51(0.44,0.59)	0.55(0.46,0.66)	0.65(0.61,0.69)	<0.001
HR (95% CI) ^b^	1	0.71(0.64,0.80)	0.65(0.57,0.74)	0.57(0.48,0.67)	0.62(0.51,0.76)	0.68(0.63,0.74)	<0.001
Deaths from cardiovascular causes % (n)	47.1(376)	27.7 (221)	16.5 (132)	5.9 (47)	2.8 (22)		<0.001
HR (95% CI) ^a^	1	0.73(0.61,0.86)	0.67(0.55,0.82)	0.39(0.29,0.54)	0.35(0.22,0.55)	0.65(0.57,0.73)	<0.001
HR (95% CI) ^b^	1	0.76(0.63,0.92)	0.73(0.57,0.92)	0.50(0.36,0.71)	0.40(0.24,0.67)	0.67(0.58,0.78)	<0.001
Deaths from myocardial infarction causes %(n)	48.3 (146)	27.2 (82)	16.9 (51)	5.6 (17)	2.0(6)		<0.001
HR (95% CI) ^a^	1	0.69(0.52,0.89)	0.63(0.45,0.88)	0.33(0.20,0.56)	0.21(0.09,0.49)	0.56(0.46,0.67)	<0.001
HR (95% CI) ^b^	1	0.77(0.57,1.04)	0.67(0.45,0.99)	0.46(0.26,0.81)	0.27(0.10,0.72)	0.60(0.47,0.75)	<0.001
Deaths from stroke causes %(n)	43.5 (133)	30.4 (93)	16.7 (51)	6.5 (20)	2.9 (9)		<0.001
HR (95% CI) ^a^	1	0.88(0.68,1.15)	0.79(0.57,1.11)	0.54(0.33,0.89)	0.51(0.25,1.04)	0.81(0.67,0.99)	0.08
HR (95% CI) ^b^	1	0.89(0.65,1.21)	0.86(0.59,1.27)	0.66(0.39,1.13)	0.55(0.25,1.21)	0.85(0.67,1.08)	0.18
Deaths from respiratory disease causes %(n)	61.1 (149)	20.9(51)	11.0 (27)	4.9 (12)	2.1 (5)		<0.001
HR (95% CI) ^a^	1	0.39(0.28,0.54)	0.29(0.19,0.44)	0.19(0.10,0.35)	0.13(0.05,0.33)	0.28(0.23,0.35)	<0.001
HR (95% CI) ^b^	1	0.43(0.30,0.62)	0.39(0.25,0.62)	0.26(0.13,0.51)	0.24(0.09,0.63)	0.35(0.27,0.44)	<0.001
Cardiovascular disease events %(n)	33.8 (1206)	25.9 (924)	18.6 (664)	12.6 (446)	9.1 (325)		<0.001
HR (95% CI) ^a^	1	0.83(0.76,0.91)	0.68(0.62,0.75)	0.54(0.48,0.61)	0.50(0.43,0.57)	0.72(0.68,0.76)	<0.001
HR (95% CI) ^b^	1	0.85(0.77,0.94)	0.75(0.67,0.84)	0.64(0.56,0.73)	0.58(0.49,0.68)	0.77(0.72,0.83)	<0.001
Myocardial infarction events %(n)	39.7 (429)	26.9(291)	17.8 (192)	8.8 (95)	6.8 (74)		<0.001
HR (95% CI) ^a^	1	0.79(0.68,0.92)	0.67(0.56,0.80)	0.43(0.34,0.54)	0.47(0.35,0.62)	0.69(0.63,0.76)	<0.001
HR (95% CI) ^b^	1	0.79(0.67,0.94)	0.70(0.57,0.85)	0.48(0.37,0.63)	0.59(0.43,0.81)	0.74(0.66,0.83)	<0.001
Stroke events %(n)	46.7 (233)	25.9 (129)	15.4 (77)	7.4 (37)	4.6 (23)		<0.001
HR (95% CI) ^a^	1	0.69(0.56,0.86)	0.62(0.47,0.81)	0.46(0.31,0.66)	0.47(0.29,0.76)	0.71(0.61,0.82)	<0.001
HR (95% CI) ^b^	1	0.68(0.53,0.88)	0.67(0.49,0.91)	0.51(0.34,0.77)	0.48(0.28,0.83)	0.74(0.62,0.89)	0.001
Respiratory disease events %(n)	40.3 (665)	23.1 (382)	16.2 (268)	11.9 (196)	8.5 (141)		<0.001
HR (95% CI) ^a^	1	0.55(0.41,0.63)	0.39(0.38,0.46)	0.30(0.25,0.36)	0.23(0.19,0.28)	0.43(0.40,0.47)	<0.001
HR (95% CI) ^b^	1	0.54(0.47,0.62)	0.40(0.34,0.47)	0.29(0.24,0.36)	0.21(0.16,0.26)	0.42(0.38,0.46)	<0.001

^a^Model 1: adjusting for age

^b^Model 2: adjustment for BMI, cigarette-smoking habit, alcohol intake, physical activity, height, education level, plasma vitamin C, social class, self-reported diabetes, respiratory disease, systolic blood pressure and plasma total cholesterol

There was a strong inverse association between increasing FEV1 quintiles and total all-cause mortality. Individuals in the top quintile i.e. those with better FEV1 had lower risk of mortality (48% in men and 55% in women) compared to subjects in the bottom quintile. There appeared to be a significant graded relationship between fatal and non-fatal cardiovascular (CV) events across the distribution of FEV1 in both men and women [men 95% confident interval (CI) (0.73, 0.81) and women 95% CI (0.63, 0.74)]. In men, deaths from cardiovascular causes, stroke and respiratory diseases were significantly associated with poorer lung function but this was not significant for deaths from myocardial infarction. In women, deaths from cardiovascular causes, myocardial infarction and respiratory disease were significantly associated with lung function but was not significant for deaths from stroke. Not unexpectedly, the risk of death due to respiratory illnesses was also significantly associated with poorer lung function and the hazard ratio was greater than for CV disease in both men and women. When analyzed as a continuous variable, for every 70 cL/s increase in FEV1, there was 28%-35% lower mortality risk from cardiovascular disease and 68%-72% lower mortality risk from respiratory disease. These associations were only slightly attenuated after multivariable adjustment.

In men, unlike the association of FEV1 with stroke mortality, baseline FEV1 level was not significantly associated with stroke incidence but was inversely associated with incident total cardiovascular disease. In women, both were significant. A greater magnitude of association between mortality due to respiratory diseases than CVD was seen in both men and women.

HRs for total all-cause mortality per increase of 70 cL/s FEV1, which was similar in the various subgroups examined, including adjustment by age, smoking status, BMI, physical activity, social class and plasma vitamin C are shown in Table 3. This was still apparent after exclusion of individuals with prevalent respiratory diseases of bronchitis and asthma or individuals who died within 5 years of follow-up (shown in Table 3). As shown in Table 3, across the observed range of FEV1 distribution in this cohort, each increase of 70 cL/s was associated with 23%-32% lower observed mortality risk in men and women over 18 years. This association was also consistent in subgroups analysis after adjustment for age, sex, smoking status, BMI, physical activity, vitamin C level, and social class.

**Table 3:** Cox multivariate-adjusted HRs for all-cause mortality in the EPIC-Norfolk 1993-2015 per 70cL/s increase in FEV1 in subgroups adjusted by age and other covariates

	Number/ Total	*HR (95%CI) per 70 cL/s increase in FEV1* ^a^	*P*	*HR (95%CI) per 70 cL/s increase in FEV1* ^b^	*P*
All	4734/19139	0.71(0.68, 0.73)	<0.001	0.74(0.71, 0.77)	<0.001
Gender					
Men	2572/8719	0.72(0.69, 0.76)	<0.001	0.77(0.73, 0.81)	<0.001
Women	2162/8258	0.67(0.62, 0.72)	<0.001	0.68(0.63, 0.74)	<0.001
Age					
<65	1661/13631	0.74(0.70, 0.78)	<0.001	0.79(0.74, 0.84)	<0.001
≥65	3073/5508	0.67(0.65, 0.71)	<0.001	0.70(0.67, 0.74)	<0.001
Excluding early deaths < 5 years	4196/18601	0.72(0.69, 0.75)	<0.001	0.76(0.72, 0.79)	<0.001
Smoking status					
-Current smokers	670/ 2214	0.72(0.65, 0.80)	<0.001	0.71(0.63, 0.79)	<0.001
-Ex-smokers	2344/7955	0.71(0.67, 0.75)	<0.001	0.72(0.68, 0.76)	<0.001
-Never smokers	1720/8970	0.81(0.75, 0.87)	<0.001	0.82(0.76, 0.89)	<0.001
By BMI					
<27 kg/m^2^	2759/12146	0.69(0.66, 0.73)	<0.001	0.73(0.69, 0.77)	<0.001
≥27 kg/m^2^	1975/6993	0.72(0.69, 0.78)	<0.001	0.75(0.71, 0.81)	<0.001
By physical activity					
- Inactive	1977/5467	0.65(0.62, 0.69)	<0.001	0.68(0.64, 0.72)	<0.001
- Active	2757/13672	0.76(0.72, 0.80)	<0.001	0.79(0.75, 0.84)	<0.001
Excluding asthma or bronchitis	4772/17837	0.72(0.69, 0.75)	<0.001	0.76(0.73, 0.79)	<0.001
Social class					
-Nonmanual social class	2737/11509	0.73(0.69, 0.77)	<0.001	0.75(0.71, 0.80)	<0.001
-Manual social class	1997/7630	0.69(0.65, 0.73)	<0.001	0.72(0.68, 0.77)	<0.001
Plasma Vitamin C					
-Less than median	2646/9206	0.71(0.67, 0.75)	<0.001	0.73(0.69, 0.77)	<0.001
-more than median	2088/9933	0.74(0.69, 0.78)	<0.001	0.75(0.71, 0.80)	<0.001

Foot note: ^a^ Model 1: adjusting for age

^b^Model 2: adjustment for BMI, cigarette-smoking habit, alcohol intake, physical activity, height, education level, plasma vitamin C, social class, self-reported diabetes, respiratory disease, systolic blood pressure and plasma total cholesterol

## Discussion

This study demonstrates that in middle-aged and older men and women, FEV1 was inversely associated with total mortality. There appears to be a graded relationship between FEV1 and mortality, with lowest mortality observed in those with higher FEV1. This inverse association was consistent after adjusting for age, BMI, height, physical activity, smoking, plasma vitamin C concentrations, occupational social class, education, systolic blood pressure, total cholesterol and diabetes. Our findings are consistent with findings from other studies [15–20]. The Renfrew and Paisley prospective population study in Scotland, [15] reported a significant inverse association between lower predicted FEV1 and all-cause mortality in both male and female after adjustment; however, participants’ height, diabetes status, bronchitis, asthma and physical activity were unaccounted for. Similarly the National Health and Nutrition Examination Survey Epidemiologic Follow-up Study from 1971 through 1992 and the Buffalo Health study (554 men and 641 women) from 1960 through 1989 showed that individuals with the lowest predicted FEV1 had the highest risk of cardiovascular death [18].

Not unexpectedly in our study, more subjects with lower FEV1 had asthma and bronchitis. The underlying pathology in asthma and bronchitis is endothelial pathogeny and persistent low-grade bronchial inflammation which has been proposed to be responsible for endothelium dysfunction and atherosclerosis, and subsequently contribute to fatal cardiovascular disease [20–25]. Smoking also has a direct effect on the development of pulmonary hypertension which in turn increases mortality and morbidity from cardiovascular related disease [26]. Patients with poorer lung function are prone to get respiratory infections due to impaired mechanism in local defence system and most of them are expected to die from respiratory failure if they have frequent acute exacerbations [27–29].

Our study shows that lung function in men is not associated with fatal myocardial infarction (MI). A possible reason is that cholesterol concentration, a risk factor which has the greatest impact on causing MI [30, 31] is the same across the quintiles of FEV1 in men. In other words, men have an equal chance of dying from myocardial infarction when they have similar cholesterol levels regardless of their lung function. And hence fatal myocardial infarction is not significant with the FEV1 distribution among men in our study.

The difference in systolic BP in men across the quintiles was smaller than for women (131-143mmHg versus 125-143mmHg). As strokes are very BP dependent [32, 33] and because of this smaller difference in BP, the stroke risk is not significantly different across the quintiles. However, FEV1 has an inverse relationship in stroke related death in men but not in women, a possible explanation for which might be the fact that women in this study have a higher vitamin C consumption (59 μmol/L versus 47 μmol/L), which works as a protective mechanism to reduce fatal stroke as our earlier observations on plasma vitamin C was inversely associated with the risk of atrial fibrillation in women but not men [34].

Lower FEV1 may be a marker of smoking but our study demonstrated that after adjusting for smoking status, lower FEV1 was significantly associated with increased total mortality among smokers, former smokers and never smokers. This finding is similar to the Copenhagen City Heart Study in Denmark [35]. Even though this study shows that there is an association between FEV1 and total cardiovascular mortality and morbidity, we should not interpret the results in terms of causality in the absence of complete causal directional acyclic graphs or conceptual framework. However, it may be considered as another surrogate marker for cardiovascular risk.

### Strength and limitation

Our present study has several strengths and some limitations. The strength of our study is that it was done in the community where the findings reflect more that of the general population at large. Secondly, our sample size is large enough to investigate the relationship in subjects who were lifelong non-smokers, free of cancer, myocardial infarction and stroke at the outset of the study. This gives a more robust picture of the relationship between lung function and total mortality and morbidity in a general population who are at a lower risk of cardiovascular disease. The Whitehall II study in United Kingdom, in a subset of 4,817 participants who had lung function measured showed similar findings. However, their sample size was relatively small and the follow-up was much shorter at 6.4 years [20]. A further strength of our study was that mortality presented using gender stratification and included adjustments for socio-economic factors and physical inactivity. Previous studies also showed a similar relationship between FEV1 and cardiovascular mortality and morbidity [36, 37]. Our study provides a more contemporary setting for this relationship while also reaffirming previous findings.

The results of this study should be interpreted in the context of its limitations. Firstly, the gold standard for measurement of FEV1 with spirometry was not used in this study. Although a handheld spirometer has good sensitivity and specificity in identifying COPD when compared with the gold standard spirometry, the accuracy of this tool for discriminating non-obstructive pulmonary abnormalities has not been established [38]. Furthermore, it may overestimate the prevalence of COPD as the cut-off point of FEV1/FVC for a handheld spirometry is higher compared with the FEV1/FVC ratio used in the gold standard spirometry [39]. Secondly, two instead of three FEV1 measurements were taken in the study. However, in view that this study is a large-scale community-based survey, it was not practical to perform three measurements or the gold standard spirometry test. Despite this limitation, the results of this study can still provide health care professionals with some insights on the association between the FEV1 and total cardiovascular mortality and morbidity. The findings in this study has shown that a portable handheld device can serve as a screening tool to identify those with abnormal results for a further formal assessment in a specialised centre. Thirdly, there was some missing data (n=596) on the FEV1 but this only represented 2% of the total population and did not affect our findings in any substantial way. Thus, the implication of the current study was that the portable handheld device can serve as a screening tool to screen for those at risk of having cardiovascular or respiratory morbidity.

## Conclusions

A lower FEV1 is an independent risk factor for all-cause and cardiovascular mortality and morbidity among men and women. In addition to the recommended BP, blood glucose and lipids measurements during routine clinical consultation or during healthcare screening, measuring the FEV1 with a portable handheld spirometry may be used as a surrogate marker for cardiovascular risk. Every effort should be made to identify those with poorer lung function even in the absence of cardiovascular disease as they are at greater risk of total and CV mortality

## Abbreviations

BMI: Body Mass Index
BP: Blood Pressure
CI: Confident interval
CV: Cardiovascular
CVD: Cardiovascular disease
EPIC-Norfolk: The Norfolk (UK) based European Prospective Investigation into Cancer
FEV1: Forced expiratory volume in one second
FVC: Forced vital capacity
HRs: Hazard Ratio
MI: Myocardial infarction
UK: United Kingdom
